# Social media and its effect on mental health: Friend or foe?

**DOI:** 10.1192/j.eurpsy.2021.2009

**Published:** 2021-08-13

**Authors:** C. Trivedi, Z. Mansuri, R. Vadukapuram, A. Reddy

**Affiliations:** 1 Research, St Davids Healthcare, Austin, United States of America; 2 Department Of Psychiatry, Boston Children’s Hospital/Harvard Medical School, Boston, United States of America; 3 Psychiatry, Icahn School of Medicine at Mount Sinai, New York, United States of America; 4 Psychiatry, Virginia Tech Carilion School of Medicine, Roanoke, United States of America

**Keywords:** Addiction, social media, Mental Health Policy, mental health

## Abstract

**Introduction:**

Recently, several studies have shown both positive and negative impacts of social media on mental health. However, little is known regarding the reasons for the negative impact of social media on mental health.

**Objectives:**

To evaluate the role of social media on mental health.

**Methods:**

We reviewed the documentary ‘The Social Dilemma’ released on Netflix in September 2020, which explored the role of social media in our life. The documentary discussed the behind the scene development of the social media world.

**Results:**

The central message from the documentary is that all the social media applications we use are capable of hijacking the thought process of your brain and are consciously designed by the artificial intelligence technology in a way that one spends more time on them. It collects users’ data such as topics they like, follow, search, subscribe, shop, and several others. Based on this data it feeds you the information according to your taste and next time you log in on the website, you spend more time on it. This causes positive reinforcement, the more time you spend on a particular topic, the more you will be presented which results in addictive behavior.

**Conclusions:**

It is known that social media addiction is prevalent, and it affects brain like drug and alcohol addiction. This documentary provided technological insight into this type of behavior. Though social media has its pros, it has numerous cons despite being used for right intentions. Better regulatory measures are needed to prevent psychological disorders related to social media usage.

**Disclosure:**

No significant relationships.

